# Low copy number of mitochondrial DNA (mtDNA) predicts worse prognosis in early-stage laryngeal cancer patients

**DOI:** 10.1186/1746-1596-9-28

**Published:** 2014-02-05

**Authors:** Siwen Dang, Yiping Qu, Jing Wei, Yuan Shao, Qi Yang, Meiju Ji, Bingyin Shi, Peng Hou

**Affiliations:** 1 Department of Endocrinology, The First Affiliated Hospital of Xi’an Jiaotong University School of Medicine, 710061 Xi’an, the People’s Republic of China; 2 Department of Otolaryngology, The First Affiliated Hospital of Xi’an Jiaotong University School of Medicine, 710061 Xi’an, the People’s Republic of China; 3 Center for Translational Medicine, The First Affiliated Hospital of Xi’an Jiaotong University School of Medicine, 710061 Xi’an, the People’s Republic of China

**Keywords:** Laryngeal cancer, Mitochondrial DNA (mtDNA), Copy number, Real-time quantitative PCR, Clinical outcomes

## Abstract

**Objectives:**

Alterations in mitochondrial DNA (mtDNA) copy number have been widely reported in various human cancers, and been considered to be an important hallmark of cancers. However, little is known about the value of copy number variations of mtDNA in the prognostic evaluation of laryngeal cancer.

**Design and methods:**

Using real-time quantitative PCR method, we investigated mtDNA copy number in a cohort of laryngeal cancers (n =204) and normal laryngeal tissues (n =40), and explored the association of variable mtDNA copy number with clinical outcomes of laryngeal cancer patients.

**Results:**

Our data showed that the relative mean mtDNA content was higher in the laryngeal cancer patients (11.91 ± 4.35 copies) than the control subjects (4.72 ± 0.70 copies). Moreover, we found that mtDNA content was negatively associated with cigarette smoking (pack-years), tumor invasion, and TNM stage. Notably, variable mtDNA content did not affect overall survival of laryngeal cancer patients. However, when the patients were categorized into early-stage and late-stage tumor groups according to TNM stage, we found that low mtDNA content was strongly associated with poor survival in the former, but not in the latter.

**Conclusions:**

The present study demonstrated that low mtDNA content was strongly correlated with some of clinicopathological characteristics, such as cigarette smoking, tumor invasion and TNM stage. In addition, we found a strong link between low mtDNA content and worse survival of the patients with early-stage tumors. Taken together, low copy number of mtDNA may be a useful poor prognostic factor for early-stage laryngeal cancer patients.

**Virtual slides:**

The virtual slides for this article can be found here: http://www.diagnosticpathology.diagnomx.eu/vs/1841771572115955

## Introduction

Laryngeal cancer represents the second most common malignancy of the head and neck worldwide [1]. Given the fundamental role the larynx plays in human speech and communication, determining the optimal management of laryngeal cancer is critical. Despite multiple and aggressive therapeutic interventions, there has been no fundamental improvement in the 5-year survival rates of the patients over the past decades [1,2]. Current methods used to predict the outcome of laryngeal cancer patients include some clinicopathological factors, including TNM stage, differentiation grade and metastasis, as well as several biomarkers, such as *hTERC* amplification and *VEGF* expression [3-11].

In recent years, copy number variations of mitochondrial DNA (mtDNA) have been reported in various human cancers, including head and neck cancer [12,13]. The major role of mitochondria, which are organelles found in all nucleated cells, is to generate cellular adenosine triphosphate (ATP) through oxidative phosphorylation [14]. Human mitochondrial DNA (mtDNA) is a 16.5-kb double-stranded DNA molecule, which contains genes coding for 13 polypeptides of the respiratory chain, 22 tRNAs and 2 rRNAs [15]. Mutations in the displacement loop (D-loop), a noncoding region essential for the replication and transcription of mtDNA, can cause a reduction in mtDNA copy number or altered mtDNA gene expression [16,17]. Cellular mtDNA content typically ranges from hundreds to more than 10 000 copies per cell, varying across different cell types. Because of lack of introns, inability to bind to histones, and inefficient mtDNA proofreading and DNA repair systems, mtDNA is more susceptible to oxidative damage than nuclear DNA (nDNA) [18]. In general, mtDNA copy number in the cells is not under stringent control; and various internal or external factors associated with ATP demand may influence its level, such as hypoxia (a strict microenvironment that carcinoma cells can proliferate fast and survive in). Until now, there have been only a few studies suggesting an increase in mtDNA copy number in laryngeal cancers as compared with normal laryngeal tissues [19]. However, the association of mtDNA content with clinical outcomes of laryngeal cancer patients remains largely unknown.

In this study, using real-time quantitative PCR method, we investigated mtDNA copy number in a large cohort of laryngeal cancer tissues, and further explored the association of mtDNA content with clinical outcomes of laryngeal cancer patients.

## Material and methods

### Patients

Informed consent was obtained from each patient according to the protocols approved by the ethics committees of the First Affiliated Hospital of Xi’an Jiaotong University School of Medicine. A total of 204 paraffin-embedded laryngeal cancer tissues and 40 paraffin-embedded noncancerous laryngeal tissues were randomly obtained at the First Affiliated Hospital of Xi’an Jiaotong University School of Medicine between January 2002 and September 2010. None of these patients had received radiotherapy or chemotherapy before surgery. The histologic diagnosis of tumors was made and agreed upon by at least two senior pathologists at Department of Pathology of the Hospital based on World Health Organization (WHO) criteria. Relevant clinicopathological data were obtained from the patients’ files or by interview with the patients or their relatives, and the details were summarized in Table 1.

**Table 1:** Clinicopathological characteristics of laryngeal cancer patients

**Characteristics**	**No. of patients (%)**
Gender	
Male	197 (96.6)
Female	7 (3.4)
Age, years	
Mean	60.75
SD	9.64
Differentiation	
Well/moderate	187 (91.7)
Poor/undifferentiation	17 (8.30)
Tumor invasion	
T1	61 (29.9)
T2	70 (34.3)
T3	57 (27.9)
T4	16 (7.8)
TNM stage	
I	56 (27.5)
II	35 (17.2)
III	74 (36.3)
IV	39 (19.1)
Lymph node metastasis (LNM)	
Yes	66 (32.4)
No	138 (67.6)
Smoking history	
Yes	141 (69.1)
No	63 (30.9)
Pack-years	
≤30	149 (73.0)
>30	55 (27.0)
Survival status	
Dead	113 (55.4)
Alive	91 (44.60)

### DNA preparation

Genomic DNA was extracted from paraffin-embedded tissues as previously described [20]. Briefly, after a treatment for 12 h at room temperature with xylene to remove paraffin, the tissues were then subjected to digestion with 1% sodium dodecyl sulfate (SDS) and proteinase K at 48°C for 48 h, with addition of several spiking aliquots of concentrated proteinase K to facilitate digestion. DNA was subsequently isolated using a standard phenol-chloroform extraction and ethanol precipitation protocol, and stored at -80°C until use.

### mtDNA copy number analysis

We measured the relative mtDNA copy number in a cohort of laryngeal cancers and normal laryngeal tissues by real-time quantitative PCR method as described previously [21]. The specific primers and TaqMan probes for *MT-ND1* and β*-actin* genes used in this study were designed using Primer Express 3.0 (Applied Biosystems, Foster City, CA) and presented in Table 2. Using a PCR protocol described previously [22], PCR amplification were carried out in a final reaction mixture of 20 μl containing 16.6 mM ammonium sulfate, 67 mM Tris base, 2.5 mM MgCl_2_, 10 mM 2-mercaptoethanol, 0.1% DMSO, 0.2 mM each of dATP, dCTP, dGTP and dTTP, 600 nM each of forward and reverse primers, 200 nM TaqMan probe, 0.6 unit Platinum *Taq* polymerase and 2% Rox reference dye. Serial dilutions of normal leukocyte DNA were used to establish standard curves. The internal reference gene β*-actin* was run in parallel to standardize the input DNA. Each sample was run in triplicate. The relative mtDNA copy number of each sample was calculated as described previously [23,24].

**Table 2:** The primer and TaqMan probe sequences used in this study

**Genes**	**Forward primer sequence**	**Probe sequence**	**Reverse primer sequence**	**Amplification**
	**(5′ → 3′)**	**(5′ → 3′)**	**(5′ → 3′)**	**Efficiency (%)**
*MT-ND1*	CCCCTAAAACCCGCCACATC	6FAM-ACCCTCTACATCACCGCCCCGACC-TAMRA	GTAGAAGAGCGATGGTGAGAGC	94.6
β*-actin*	TCACCCACACTGTGCCCATCTACGA	6FAM-ATGCCCTCCCCCATGCCATCC-TAMRA	TCGGTGAGGATCTTCATGAGGTA	95.2

### Statistical analysis

The copy number of mtDNA between laryngeal cancer and normal laryngeal tissues were compared by the Mann–Whitney *U* test. Association of mtDNA copy number with clinicopathological characteristics was univariately assessed using the SPSS statistical package (version 11.5, Chicago, IL). Multivariate models were then developed that adjusted for the most important covariates, including gender, age, smoking history, and TNM stage. The day of primary tumor surgery to the day of death or last clinical follow-up was used to determine the survival length. The Kaplan–Meier method was used for survival analysis grouping with mtDNA copy number. Differences between curves were analyzed using the log-rank test. Multivariate Cox regression analysis was employed to evaluate the impact of variable mtDNA copy number on survival of independently of the number of lymph node metastasis, tumor invasion and differentiation. All statistical analyses were performed using the SPSS statistical package (version 11.5, Chicago, IL). *P* values < 0.05 were considered significant.

## Results

### Relative mtDNA copy number in laryngeal cancer

**Figure 1:**
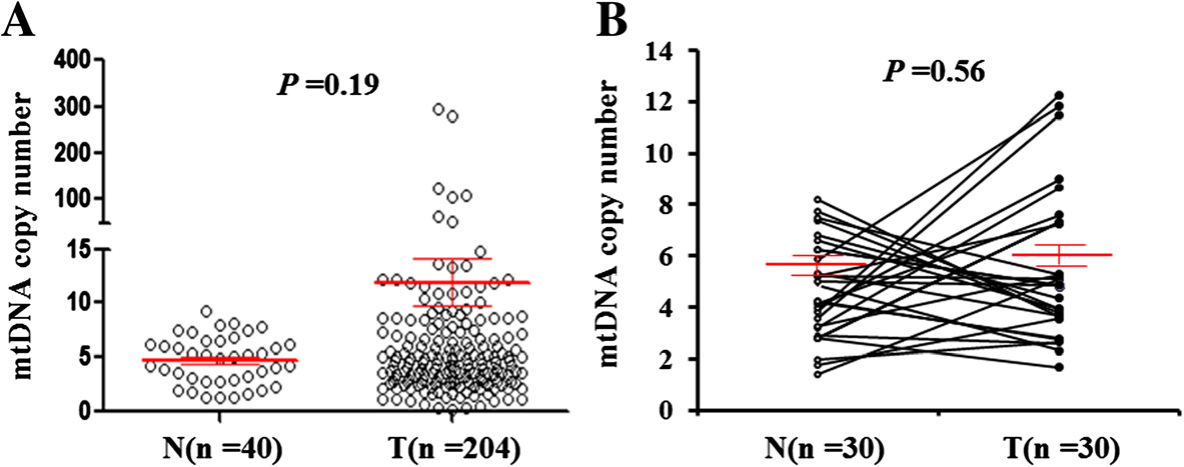
**Copy number analysis of mtDNA in laryngeal cancer. (A)** Copy number of mtDNA corresponding to each individual case of laryngeal cancers and normal laryngeal tissues (circle). Real-time quantitative PCR assay was performed to analyze mtDNA copy number in a cohort of laryngeal cancers and normal laryngeal tissues. Details are as described in Methods. **(B)** Copy number of mtDNA in 30 pairs of laryngeal cancer tissues and their corresponding normal tissues by real-time quantitative PCR. Horizonal lines represent mean ± S.E. N, normal laryngeal tissues; T, laryngeal cancer tissues.

We investigated mtDNA copy number in a total of 204 laryngeal cancers and 40 normal laryngeal tissues using real-time quantitative PCR assay. As shown in Figure 1A, the relative mean mtDNA content was higher in laryngeal cancer patients (11.91 ± 4.35 copies) than control subjects (4.72 ± 0.70 copies), which was consistent with a previous study [16]. However, the difference did not reach statistical significance (*P* =0.19). The median values among laryngeal cancer patients and control subjects were 4.92 copies (range =0.22-293.54 copies) and 4.92 copies (range =1.28-9.21 copies), respectively. Moreover, a total of 30 pairs of laryngeal cancer tissues and their corresponding normal tissues were compared using Wilcoxon Signed Ranks test. The data showed that there was not significant difference in relative mean mtDNA copy number between cancer and normal tissues (*P* =0.56) (Figure 1B).

**Figure 2:**
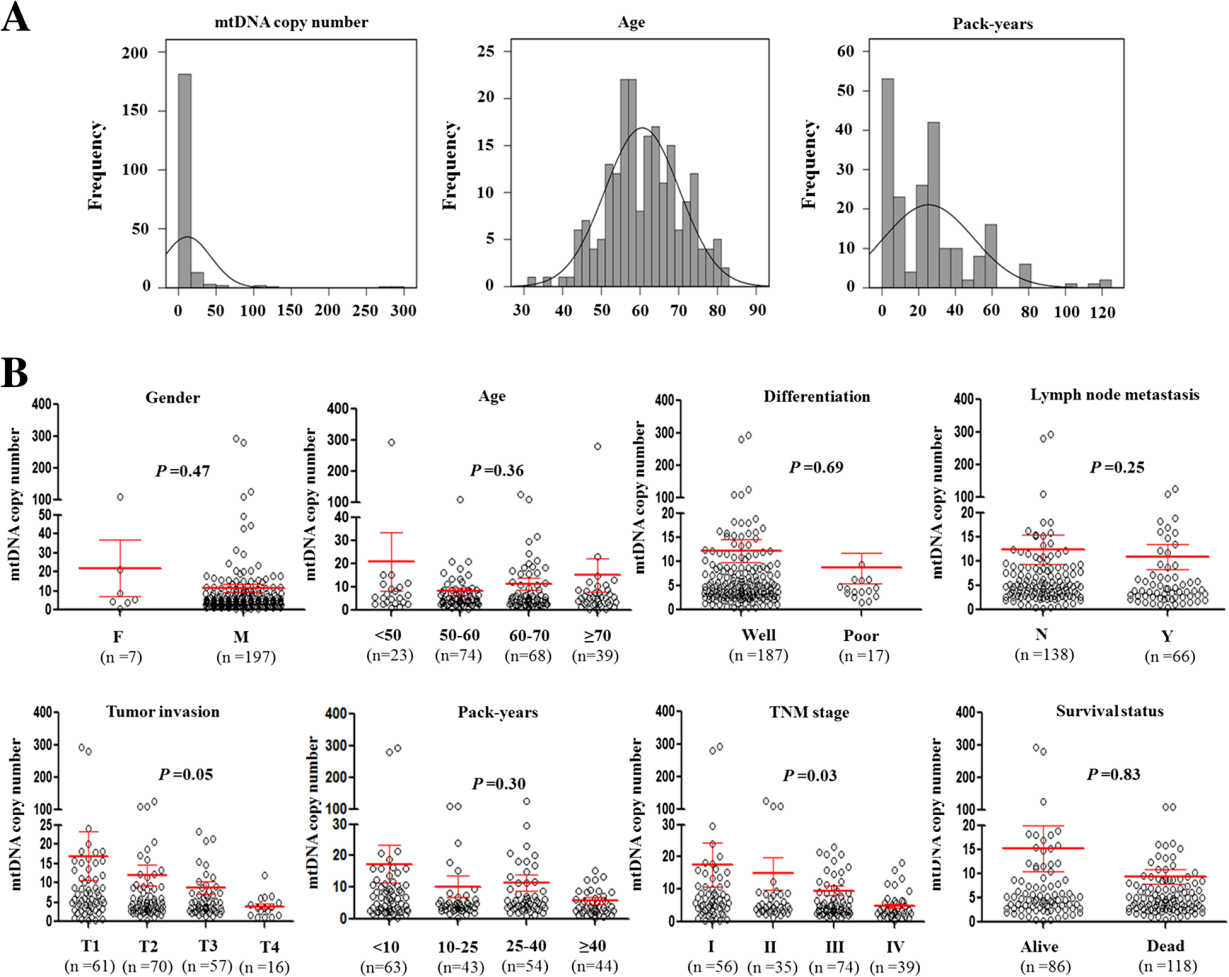
**Association of mtDNA copy number with clinicopathological variables in laryngeal cancer.** Copy number of mtDNA was analyzed using real-time quantitative PCR approach. Details are as described in Methods. **(A)** A frequency distribution of the number of cases by mtDNA copy number, age, and pack-years. **(B)** The relationship of mtDNA copy number with various clinical features of laryngeal cancer patients. The circle represents mtDNA copy number of each case of laryngeal cancers. Horizonal lines represent mean ± S.E. Sample means were compared using the Mann–Whitney *U* test. F, female; M, male; Well, well/moderate differentiation; Poor, poor/undifferentiation; N, non-lymph node metastasis; Y, lymph node metastasis.

We next evaluated whether mtDNA copy number differed by selected clinicopathological characteristics. As shown in Figure 2B, heavy-smoking patients had a lower mtDNA copy number than those with mild or without smoking history (<10 pack- years: 17.39 copies; 10–25 pack-years: 10.23 copies; 25–40 pack-years: 11.51 copies; ≥40 pack-years: 5.78 copies, *P* =0.30). Also shown in Figure 2B, the tumors with low mtDNA content were more aggressive as compared with those with high mtDNA content (T1: 16.92 copies; T2: 11.92 copies; T3: 8.64 copies; T4: 3.96 copies; *P* =0.05). In addition, the patients with late-stage tumors had a remarkable lower mtDNA content than those with early-stage tumors (stage I: 17.61 copies; stage II: 14.91 copies; stage III: 9.59 copies; stage IV: 4.97 copies; *P* =0.03), especially stage IV. Moreover, we did not find significant associations of mtDNA copy number with other clinicopathological features, such as gender, age, differentiation, lymph node metastasis and survival status (Figure 2B).

### Association of variable mtDNA copy number with clinicopathological characteristics of laryngeal cancer patients

To further investigate the relationship of mtDNA content with clinicopathological characteristics of laryngeal cancer patients, we chosed two cutoff points, which are the lower and upper limit (4.02 and 5.42 copies) of the overall 95% confidence interval for all control subjects, respectively. Laryngeal cancer patients were then categorized into three groups by use of these two cutoff points, including individuals with highest (>5.42 copies) (termed “high mtDNA content” hereafter), medium (4.02-5.42 copies) and lowest (<4.02 copies) (termed “low mtDNA content” hereafter) category of mtDNA content. Medium category of mtDNA content (4.02-5.42 copies) was used as a reference. As shown in Table 3, mtDNA copy number did not significantly correlate with most of clinicopathological characteristics. However, low mtDNA content was found to be significantly associated with TNM stage (OR =1.53, 95% CI =1.04-2.25; *P =*0.03), as compared with the reference. Also shown in Table 3, high mtDNA content was significantly negatively associated with smoking history (at least 10 pack-years of smoking) (OR =0.29, 95% CI =0.10-0.84; *P* =0.02). Although no statistical significance was noted, there was a trend toward association of low mtDNA content with gender (OR =2.73, 95% CI =0.37-20.28; *P* =0.33) and lymph node metastasis (OR =2.31, 95% CI =0.90-5.95; *P* =0.08), respectively (Table 3).

**Table 3:** Copy number variations of mtDNA in laryngeal cancer — univariate associations with clinicopathological characteristics

**Characteristics**	**Copy number <4.02**		**Copy number >5.42**	
	OR* (95% CI)	***P***	OR* (95% CI)	***P***
Male *vs.* Female	2.73 (0.37-20.28)	0.33	1.89 (0.30-11.86)	0.50
Age	1.00 (0.96-1.05)	0.92	1.01 (0.97-1.05)	0.78
Differentiation^1^	1.02 (0.25-4.10)	0.98	0.64 (0.17-3.01)	0.64
Tumor invasion^2^	1.43 (0.92-2.25)	0.11	0.94 (0.60-1.46)	0.77
TNM stage^3^	1.53 (1.04-2.25)	0.03	1.04 (0.71-1.51)	0.84
LNM^4^	2.31 (0.90-5.95)	0.08	1.50 (0.58-3.89)	0.41
Smoking history^5^	0.46 (0.16-1.34)	0.16	0.29 (0.10-0.84)	0.02
Survival status^6^	0.81 (0.36-1.84)	0.61	0.79 (0.35-1.77)	0.56

*OR: odds ratio with 95% confidence interval; ^1^Differentiation (well or moderate; poor or undifferentiation); ^2^Tumor invasion (T1; T2; T3; T4); ^3^TNM stage (I; II; III; IV); ^4^LNM (lymph node metastasis); ^5^Smoking history (at least 10 pack-years); ^6^Survival status (Alive *vs.* Dead); The cases with 4.02-5.42 mtDNA copies were used as reference.

Laryngeal cancer patients were further categorized into two groups based on TNM stage, such as individuals with early-stage (stages I and II) and late-stage (stages III and IV) tumors. Given that most of the cases with early-stage tumors are male (97.8%) and well differentiation (94.5%), we did not investigate the associations of mtDNA content with these two clinicopathological variables. As shown in Table 4, high mtDNA content was significantly negatively associated with smoking history in the patients with early-stage tumors (OR =0.17, 95% CI =0.04-0.85, *P* =0.03). However, we did not find the associations of variable mtDNA content with the other characteristics in laryngeal cancer patients. In addition, our data showed that variable mtDNA content did not significantly correlate with any clinicopathological variables in the patients with late-stage tumors (Table 5). To assess the independent association of mtDNA content with gender, age, smoking history and TNM stage, we conducted multiple multivariable logistic regressions. As shown in Table 6, similar to univariate analysis*,* low mtDNA content remained significantly associated with TNM stage (OR =1.62, 95% CI =1.09-2.41; *P* =0.02). Moreover, high mtDNA content remained negatively associated with smoking history (OR =0.27, 95% CI =0.09-0.80; *P* =0.02) (Table 6).

**Table 4:** Copy number variations of mtDNA in early-stage laryngeal cancer — univariate associations with clinicopathological characteristics

**Characteristics**	**Copy number <4.02**		**Copy number >5.42**	
	OR^* ^**(95% CI)**	***P***	OR^* ^**(95% CI)**	***P***
Age	1.00 (0.94-1.06)	0.93	1.03 (0.98-1.09)	0.29
Tumor invasion^1^	1.25 (0.39-4.04)	0.71	0.48 (0.15-1.52)	0.21
Smoking history^2^	0.25 (0.05-1.31)	0.10	0.17 (0.04-0.85)	0.03
Survival status^3^	1.09 (0.34-3.54)	0.88	0.54 (0.17-1.69)	0.29

*OR: odds ratio with 95% confidence interval; ^1^Tumor invasion (T1; T2; T3; T4); ^2^Smoking history (at least 10 pack-years); ^3^Survival status (Alive *vs.* Dead); The cases with 4.02-5.42 mtDNA copies were used as reference.

**Table 5:** Copy number variations of mtDNA in late-stage laryngeal cancer — univariate associations with clinicopathological characteristics

**Characteristics**	**Copy number <4.02**		**Copy number >5.42**	
	OR^* ^**(95% CI)**	***P***	OR^* ^**(95% CI)**	***P***
Male *vs.* Female	8.83 (0.74-105.58)	0.09	3.58 (0.46-28.17)	0.23
Age	0.99 (0.93-1.06)	0.76	0.97 (0.91-1.04)	0.37
Differentiation^1^	0.37 (0.08-1.81)	0.22	0.36 (0.07-1.84)	0.22
Tumor invasion^2^	1.05 (0.48-2.33)	0.90	0.78 (0.35-1.74)	0.54
LNM^3^	1.57 (0.48-5.12)	0.45	1.37 (0.41-4.56)	0.61
Smoking history^4^	0.78 (0.19-3.21)	0.73	0.49 (0.12-2.04)	0.33
Survival status^5^	0.64 (0.20-2.08)	0.45	1.05 (0.32-3.47)	0.94

*OR: odds ratio with 95% confidence interval; ^1^Differentiation (well or moderate; poor or undifferentiation); ^2^Tumor invasion (T1; T2; T3; T4); ^3^LNM (lymph node metastasis); ^4^Smoking history (at least 10 pack-years); ^5^Survival status (Alive *vs.* Dead); The cases with 4.02-5.42 mtDNA copies were used as reference.

**Table 6:** Copy number variations in laryngeal cancer — multivariable models assessing gender, age, smoking history, and TNM stage

**Characteristics**	**Copy number <4.02**		**Copy number >5.42**	
	OR^* ^**(95% CI)**	***P***	OR^* ^**(95% CI)**	***P***
Gender	3.48 (0.46-26.59)	0.23	2.45 (0.37-16.42)	0.36
Age, years	1.00 (0.96-1.04)	0.96	1.00 (0.96-1.05)	0.87
Smoking history^1^	0.39 (0.13-1.17)	0.09	0.27 (0.09-0.80)	0.02
TNM stage^2^	1.62 (1.09-2.41)	0.02	1.12 (0.76-1.65)	0.57

*OR: odds ratio with 95% confidence interval; ^1^Smoking history (at least 10 pack-years); ^2^TNM stage (I; II; III; IV); The cases with 4.02-5.42 mtDNA copies were used as reference.

### Impact of variable mtDNA content on poor survival of laryngeal cancer patients

**Figure 3:**
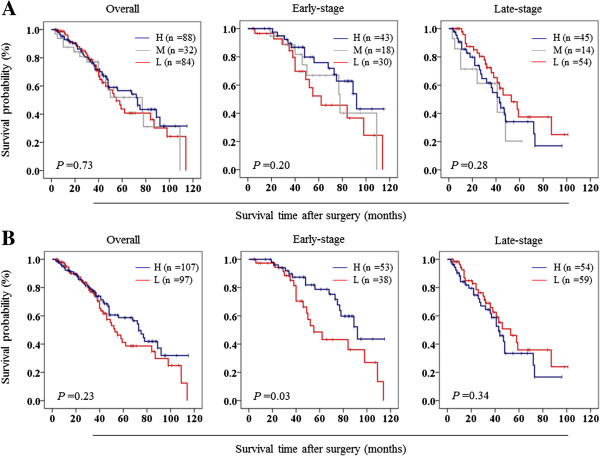
**The effect of variable mtDNA content on poor survival of laryngeal cancer patients.** The lower and upper limit (4.02 and 5.42 copies) of the overall 95% confidence interval for all control subjects **(A)** and the relative mean mtDNA copy number (4.72 copies) of all control subjects **(B)** were set as the cutoff points, respectively. Kaplan-Meier analysis of survival was then performed according to copy number variations of mtDNA in a large cohort of laryngeal cancers. The results showed that both low and high mtDNA content were not associated with overall survival of the patients. However, when the data were stratified further based on the TNM tumor stage, low mtDNA content was strongly associated with worse survival in the patients who had early-stage tumors, but not in those with late-stage tumors. H, high mtDNA content; M, medium mtDNA content (or reference); L, low mtDNA content.

Whether variable mtDNA content is associated with poor survival of laryngeal cancer patients, as suggested by its association with some of clinicopathological characteristics, was then evaluated in this study. Similarly, medium category of mtDNA content (4.02-5.42 copies) was used as a reference. As shown in Table 7, variable mtDNA content did not affect overall survival of laryngeal cancer patient. Next, we used Kaplan-Meier survival curves to further determine the effect of variable mtDNA content on the survival of laryngeal cancer patients. Similar to the findings in Table 7, variable mtDNA content did not significantly affect the survival of laryngeal cancer patients (39.4 months for low mtDNA content *vs.* 43.0 months for median mtDNA content *vs.* 44.1 months for high mtDNA content on average, *P* =0.73) when the patients were divided into low (≤4.02), median (4.02-5.42), and high (>5.42) mtDNA content groups (Figure 3A). Cox multivariate repression showed that low or high mtDNA content (the former: HR =1.78, 95% CI =0.93-3.42, *P* =0.09; the latter: HR =1.40, 95% CI =0.84-2.35, *P* =0.20) is not a predictor of poor survival for laryngeal cancer patients as an independently variable with respect to lymph node metastasis, tumor invasion and differentiation. The data were stratified further based on the TNM tumor stage, because it is an independent risk factor for laryngeal cancer patients. Also shown in Figure 3A, although the difference did not reach statistically significant, low mtDNA content was associated with worse survival in the patients with early-stage tumors as compared with medium and high mtDNA content (47.1 months *vs.* 55.6 months and 57.9 months on average, *P* =0.20). However, variable mtDNA content did not affect the survival of the patients with late-stage tumors (35.1 months for low mtDNA content *vs.* 26.8 months for medium mtDNA content *vs.* 30.9 months for high mtDNA content on average, *P* =0.28). To further determine the effect of variable mtDNA content on poor survival, the patients were divided into low (≤4.72) and high (>4.72) copy number groups by using relative mean mtDNA copy number (4.72 copies) of all control subjects as cutoff point. As shown in Figure 3B, the patients with low mtDNA content had significantly shorter survival time than those with high mtDNA content in the early-stage tumors (47.9 months *vs.* 58.2 months on average, *P* =0.03). Similarly, high mtDNA content was not associated with poor survival of the patients with late-stage tumors (Figure 3B).

**Table 7:** Prognostic value of clinicopathological factors and copy number variation of mtDNA in univariate and multivariate Cox regression analysis (n =204)

	**Univariate analysis**		**Multivariate analysis**	
**Variable**	**Hazard Ratio (95% CI)**	***P***	**Hazard Ratio (95% CI)**	***P***
Copy number				
4.02 ~ 5.42	1.00 (reference)		1.00 (reference)	
<4.02	1.00 (0.54–1.83)	0.99	1.78 (0.93–3.42)	0.09
>5.42	0.84 (0.53–1.34)	0.46	1.40 (0.84–2.35)	0.20
Lymph node metastasis				
No	1.00 (reference)		1.00 (reference)	
Yes	2.70 (1.71–4.25)	<0.001	2.43 (1.46–4.05)	0.001
Tumor invasion				
T1	1.00 (reference)		1.00 (reference)	
T2	1.98 (1.15–3.43)	0.015	1.71 (0.95–3.06)	0.07
T3	2.32 (1.29–4.18)	0.005	2.24 (1.22–4.14)	0.01
T4	5.46 (2.28–13.11)	<0.001	5.42 (2.08–14.13)	0.001
Differentiation				
Well/moderate	1.00 (reference)		1.00 (reference)	
Poor/undifferentiation	2.78 (1.10–7.07)	0.03	2.33 (0.88–6.15)	0.09

## Discussion

Given the essential involvement of mitovhondria in cellular bioenergetic and in many important physiological processes, including metabolism, signaling, apoptosis, cell cycle, and differentiation, it is not surprising that mitochondria dysfunction can contribute to the development of various human disease, inculding cancers [25,26]. Unlike nuclear DNA, mtDNA is present at a consistently high level in normal cells [27], and the mitochondrial genome lacks introns and protective histones. As a consequence, the mutation rate of mtDNA is substantially greater than that of nuclear genomic DNA [26,28]. It has been well documented that excess reactive oxygen species (ROS) acts not only mutagens and initiators of oxidative stress, but are also significant inter- and intra-cellular signaling molecules, contribute to a number of nuclear and mitochondrial changes in gene expression [29,30]. Moreover, mitochondrial has been reported to be highly susceptible to ROS [31]. ROS is thus often considered as an important determinant of cancer risk.

Mitochondrial aberrants, including mtDNA somatic mutations and copy number variations, have been frequently reported in various human cancers [13,21,24,32-35], including laryngeal cancer [19]. However, the prognostic value of copy number variations of mtDNA in laryngeal cancer patients remains to be explored. In this study, we investigated relative mtDNA copy number in a cohort of laryngeal cancers and normal larygeal tissues (control subjects) by using real-time quantitative PCR method. Our data showed that relative mean mtDNA content was higher in laryngeal cancer patients than control subjects. In line with this finding, a previous study has reported the increased mtDNA copy number in laryngeal cancer tissues as compared with paracancerous normal tissues, and demonstrated that mtDNA copy number in the cases which carried D-loop mutations was significantly higher than that of the negative cases [19]. These observatiosns suggest that the increase in mtDNA copy number, together with a high frequence of mtDNA mutations or polymorphisms in D-loop region, may play a key role in larynx carcinogenesis. Moreover, we did not find the associations of mtDNA content with gender, age, differentiation, and survival status. However, our data demonstrated that mtDNA copy number was significantly negatively associated with cigarette smoking, as supported by a previous study that cigarette smoking could modulate the mtDNA content in a negative manner in the lung tissues of the smokers [36]. We also found that low copy number of mtDNA was significantly positively associated with tumor invasion depth. In addition, our data showed that mtDNA copy number was closely associated with TNM stage. The patients with late-stage tumors had a significant lower mtDNA copy number than those with early-stage tumors. These findings suggest that variable mtDNA content, particularly low mtDNA content, may contribute to poor prognosis of laryngeal cancer patients.

To further explore the association of mtDNA content with clinical outcomes of laryngeal cancer patients, we categorized the patients into three groups by using two cutoff points (the lower and upper limit of 95% confidence interval for all control subjects), including low mtDNA content (<4.02 copies), medium mtDNA content or reference (4.02-5.42 copies) and high mtDNA content (>5.42 copies). Our data showed that mtDNA content was significantly negatively associated with TNM stage in laryngeal cancer patients, as supported by a previous study that positive correlation was found with decrease in mtDNA content with the increase in tumor stages in oral cancer [37]. Moreover, our data also showed that low mtDNA content was closely associated with an increased risk of lymph node metastasis for laryngeal cancer patients as compared to reference. When the patients were further categorized into early-stage and late-stage groups based on TNM stage, a significantly negative relationship between mtDNA content and smoking history was only found in the patient with early-stage tumors, but not in those with late-stage tumors. These observations suggest that copy number variations of mtDNA may be invloved in laryngeal cancer progression. Next, we investigated the effect of variable mtDNA content on poor survival of laryngeal cancer patients. The data showed that both low and high mtDNA content were not associated with overall survival of cancer patients. However, further analysis revealed that low mtDNA content was closely associated with poor survival only in the patients with early-stage tumors, but not in those with late-stage tumors. These data implicate that low mtDNA content can predict worse survival in the early-stage laryngeal cancer patients, as supported by a previous study that low mtDNA copy number may result in a stronger tolerance to hypoxia, and make tumor cells reduce the dependence of mitochondrial oxidative phosphorylation and get the energy for tumor progression mainly from anaerobic metabolism, further contribute to tumor cell invasion and survival under hypoxic conditions [38].

In conclusion, we investigated relative mtDNA content in a large cohort of laryngeal cancers, and demonstrated that mtDNA content was negatively associated with cigarette smoking, tumor invasion and TNM stage. In addition, low mtDNA content predicts worse survival for the patients with early-stage tumors. Therefore, variable mtDNA content may be used as a valuable biomarker to evaluate clinical outcomes of early-stage laryngeal cancer patients.

## Abbreviations

mtDNA: Mitochondrial DNA; ATP: Adenosine triphosphate; D-loop: Displacement loop; tRNA: Transfer RNA; rRNA: Ribosomal RNA; nDNA: Nuclear DNA; WHO: World Health Organization; SDS: Sodium dodecyl sulfate; MT-ND1: Mitochondrially encoded NADH dehydrogenase 1; ROS: Reactive oxygen species.

## Competing interests

The authors declare that they have no competing interests.

## Authors’ contributions

PH conceived and designed the experiments. SD, YQ and JW performed the experiments. YS, QY and MJ collected the samples and analyzed the data. BS and PH contributed reagents/materials/analysis tools. PH Wrote the paper. All authors are in agreement with the content of the manuscript and this submission.
